# Effects of prostaglandin E_2 _on the electrical properties of thermally classified neurons in the ventromedial preoptic area of the rat hypothalamus

**DOI:** 10.1186/1471-2202-6-14

**Published:** 2005-02-27

**Authors:** Heather J Ranels, John D Griffin

**Affiliations:** 1 Department of Biology, College of William and Mary. Williamsburg, Virginia 23187, USA

## Abstract

**Background:**

Physiological and morphological evidence suggests that activation of the ventromedial preoptic area of the hypothalamus (VMPO) is an essential component of an intravenous LPS-dependent fever. In response to the endogenous pyrogen prostaglandin E_2 _(PGE_2_), the majority of temperature insensitive neurons in the VMPO show an increase in firing rate, while warm sensitive neurons are inhibited. We have hypothesized that these PGE_2 _dependent effects on firing rate are due to changes in the inherent electrical properties of VMPO neurons, which are regulated by the activity of specific ionic currents.

**Results:**

To characterize the electrical properties of VMPO neurons, whole-cell recordings were made in tissue slices from male Sprague-Dawley rats. Our results indicate that PGE_2 _dependent firing rate responses were not the result of changes in resting membrane potential, action potential amplitude and duration, or local synaptic input. However, PGE_2 _reduced the input resistance of all VMPO neurons, while increasing the excitability of temperature insensitive neurons and decreasing the excitability of warm sensitive neurons. In addition, the majority of temperature insensitive neurons responded to PGE_2 _with an increase in the rate of rise of the depolarizing prepotential that precedes each action potential. This response to PGE_2 _was reversed for warm sensitive neurons, in which the prepotential rate of rise decreased.

**Conclusion:**

We would therefore suggest that PGE_2 _is having an effect on the ionic currents that regulate firing rate by controlling how fast membrane potential rises to threshold during the prepotential phase of the action potential.

## Background

Fever, an elevation in body temperature, is thought to play an adaptive role in the immune system's ability to fight infection [1]. A suggested mechanism for its production and maintenance is a shifting of the thermostatic set-point into the hyperthermic range [1,2]. Through the integration of both central and afferent thermal information, this set-point is established by the activity of neurons in the preoptic and anterior regions of the hypothalamus (PO/AH) that can be thermally classified on the basis of their inherent ability to respond to changes in temperature [3]. The majority of PO/AH neurons are considered temperature insensitive, showing little or no temperature dependent changes in firing rate. Approximately 30% of PO/AH neurons can be classified as warm sensitive, responding to local warming with an increase in firing rate [4]. While there has been considerable debate as to the criteria that should be used to classify a neuron as warm sensitive, we have used a regression coefficient of at least 0.8 impulses·s^-1^·°C^-1^. This criterion is based on previous studies that indicate a functional difference for neurons which show this degree of inherent thermosensitivity [3,4]. In addition to responding to local changes in temperature, some of these warm sensitive neurons are also responsive to changes in skin or spinal temperature, while others show thermally dependent changes in their firing rates that may directly correlate with the activation of specific thermoregulatory responses. Although this integrative ability seems to be restricted to warm sensitive neurons in the PO/AH, temperature insensitive neurons may also play an important role in determining the set-point temperature through their synaptic interactions with thermoregulatory effector neurons [3]. Regardless of thermosensitivity, many PO/AH neurons may respond to adjustments in other homeostatic conditions or the presence of endogenous pyrogens such as prostaglandin E_2 _(PGE_2_), which could shift the thermostatic set-point and alter the activation of thermoregulatory mechanisms [2].

In response to stimulation of the immune system, changes in the activity of neurons in specific regions of the PO/AH may be responsible for the adjustment of the thermostatic set-point that results in an elevation in body temperature. Physiologic evidence suggests that in response to endotoxins such as lipopolysaccharide (LPS), this shift in set-point is mediated by the activation of afferent neural pathways or the production of systemic pyrogens, which ultimately leads to the local production of PGE_2 _within the hypothalamus [5,6]. Early microinjection studies clearly established a role for prostaglandins in the production of a fever and later identified the importance of the region surrounding the OVLT in this response [7-10]. More recently, it has been shown that fever in response to intravenous LPS is dependent on the presence of the PGE_2 _producing enzyme cyclooxygenase-2 in the ventromedial preoptic area of the hypothalamus (VMPO) [11]. In addition, it has now been demonstrated that unlike other regions of the PO/AH, PGE_2 _has a selective effect on the firing rates of VMPO neurons, based on thermosensitivity, with PGE_2 _increasing the firing rates of temperature insensitive neurons and inhibiting the firing rates of warm sensitive neurons [12]. Anatomical studies also support the importance of the VMPO in the production of a fever, demonstrating that either the intravenous injection of LPS or microinjection of PGE_2 _directly into the VMPO will produce a fever that can be correlated with an increase in the cellular activation of neurons within the VMPO [13,14].

Using a functional criterion for determining the thermosensitivity of hypothalamic neurons, a clear difference in the effects of PGE_2 _on the firing rates of VMPO neurons has been demonstrated [12]. Based on current models of set-point temperature regulation, this PGE_2 _dependent increase in the firing rates of temperature insensitive neurons or decrease in the firing rates of warm sensitive neurons could lead to a hyperthermic shift in the thermostatic set-point and production of a fever [3]. Yet, little is known about the electrical responses by which PGE_2 _regulates the firing rates of VMPO neurons. We have hypothesized that these PGE_2 _dependent changes in firing rate are not the result of a change in the frequency of synaptic input to these neurons, but a selective effect on specific electrical properties of VMPO neurons. To characterize these responses, whole-cell recordings were made from VMPO neurons in tissue slices from male Sprague-Dawley rats, in response to changes in temperature and PGE_2_.

## Results

**Figure 1:**
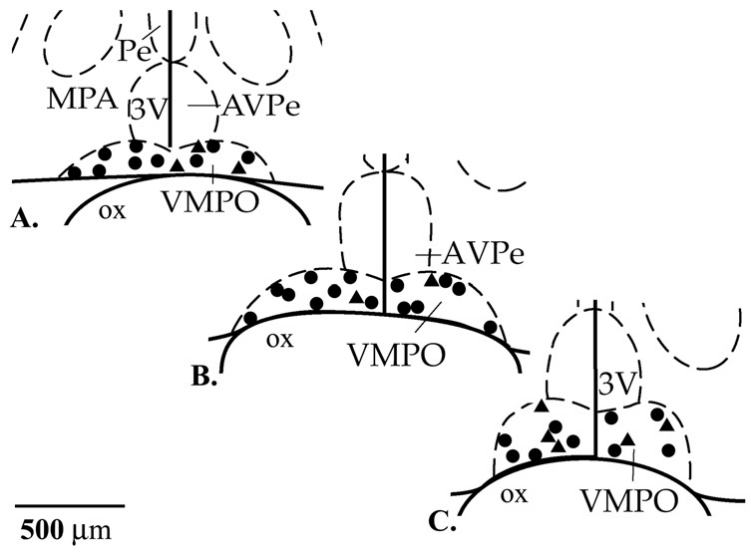
**Intracellular whole-cell recordings from VMPO Neurons. **The positions of all recorded neurons in this study are indicated on the above coronal diagrams (temperature insensitive = ●, warm sensitive = ▲ modified from Figures 18–20, Paxinos & Watson, 1998). **A **corresponds to the anterior regions of VMPO, approximately 400 μm rostral to the areas shown in **B**, which correspond to the midpoint of the VMPO. **C **corresponds to the posterior regions of VMPO, approximately 400 μm caudal to the areas shown in **B**. 3 V, third ventricle; AVPe, anteroventral periventricular nucleus; MPA, medial preoptic area; Pe, periventricular hypothalamic nucleus; ox, optic chiasm; VMPO, ventromedial preoptic nucleus.

For forty two VMPO neurons, temperature sensitivity and PGE_2 _dependent changes in firing rate, electrical activity, and the frequency of synaptic input were determined. The majority of these neurons were classified as temperature insensitive (n = 32). The remaining ten neurons were classified as warm sensitive. With respect to thermosensitivity or responses to PGE_2_, there was no specific pattern to the distribution of these neurons throughout the VMPO (Fig. 1).

Using the cellularly invasive procedure of whole cell recording, the PGE_2 _dependent changes in firing rate that were recorded from VMPO neurons were similar to those reported in an earlier extracellular single-unit recording study [12]. In response to PGE_2_, fourteen temperature insensitive neurons showed significant increases in firing rate. Thirteen of these neurons had thermosensitivities ≤ 0.4 impulses·s·°C^-1^. The firing rates of temperature insensitive neurons, having thermosensitivities ≤ 0.4 impulses·s^-1.^°C^-1^, significantly increased in response to PGE_2_, from 5.75 ± 1.31 impulses·s^-1 ^to 6.5 ± 1.31 impulses·s^-1 ^(paired T test, P = 0.04; firing rates at least 10 minutes into the following washout period = 6.37 ± 1.32 impulses·s^-1^). Of the thirteen temperature insensitive neurons with thermosensitivities of 0.41 – 0.79 impulses·s^-1.^°C^-1^, the majority (n = 10) showed little or no change in firing rate in response to PGE_2_. The firing rates of these neurons did not significantly change from a baseline of 8.4 ±1.21 impulses·s^-1 ^(PGE_2 _= 8.55 ± 1.24 impulses·s^-1 ^(paired T test, P = 0.38); washout = 8.34 ± 1.24 impulses·s^-1^). In contrast to the responses of temperature insensitive neurons, the majority (n = 8) of VMPO warm sensitive neurons showed a significant decrease in firing rate during perfusion with PGE_2_. The firing rates of warm sensitive neurons significantly decreased from 15.28 ± 4.93 impulses·s^-1 ^to 12.35 ± 4.85 impulses·s^-1 ^in response to PGE_2 _(paired T test, P = 0.003; firing rates at least 10 minutes into the following washout period = 12.87 ± 4.12 impulses·s^-1^).

### Electrical properties

All VMPO neurons recorded in this study had resting membrane potentials of -45.0 ± 1.1 mV (n = 42). There was no significant difference between the resting membrane potentials of temperature insensitive neurons (-45.6 ± 1.2 mV; n = 32) and warm sensitive neurons (-43.1 ± 2.4 mV; n = 10). In addition, resting membrane potential did not change in response to PGE_2 _and was not responsible for PGE_2 _dependent changes in firing rate.

**Figure 2:**
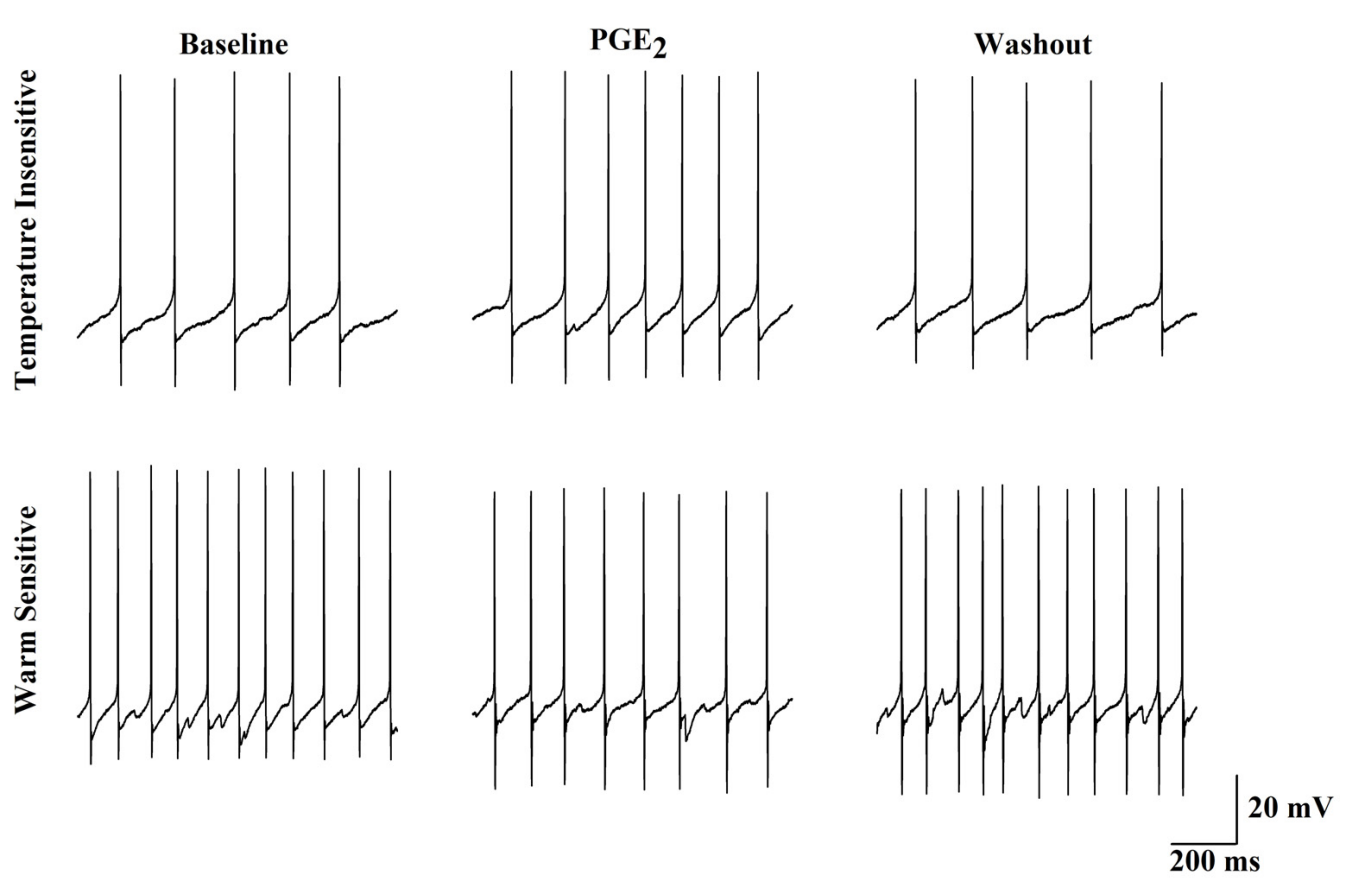
**The effects of temperature and PGE_2 _on the activity of a VMPO temperature insensitive neuron and a VMPO warm sensitive neuron. **The top panels show one second records of the action potential activity of a VMPO temperature insensitive neuron during all three experimental conditions (resting membrane potential = -43.94 mV). The firing rate significantly increased from a baseline of 5.11 ± 0.12 impulses·s^-1 ^to 7.33 ± 0.13 impulses·s^-1 ^during perfusion with 1 μM PGE_2 _(washout = 4.90 ± 0.14 impulses·s^-1^). The lower panels show one second records of the action potential activity of a VMPO warm sensitive neuron during all three experimental conditions (resting membrane potential = -51.62 mV). The firing rate significantly decreased from a baseline of 10.07 ± 0.13 impulses·s^-1 ^to 8.40 ± 0.12 impulses·s^-1 ^during perfusion with 1 μM PGE_2 _(washout = 10.20 ± 0.16 impulses·s^-1^).

The top panels of Figure 2 show the action potential activity of a temperature insensitive neuron during baseline conditions, perfusion with PGE_2_, and the washout period. While the resting membrane potential did not change from a baseline mean of -43.94 mV, firing rate increased 43.4% in response to PGE_2_, from a mean of 5.11 impulses·s^-1 ^to 7.33 impulses·s^-1^. The onset of this response occurred several minutes after perfusion with PGE_2 _had begun and lasted approximately 15 minutes beyond the point when perfusion with PGE_2 _ended. This was typical of all temperature insensitive neurons which had a significant change in firing rate in response to PGE_2_. These neurons showed response latencies of 3.5 ± 0.69 minutes and durations that ranged from 7 to 40 minutes before firing rate returned towards the baseline level.

The lower panels of Figure 2 show the action potential activity of a warm sensitive neuron during baseline conditions, perfusion with PGE_2_, and the washout period. While the resting membrane potential did not change from a baseline mean of -51.62 mV, the firing rate of this neuron decreased in response to PGE_2_, from a mean of 10.07 impulses·s^-1 ^to 8.40 impulses·s^-1^. The onset of this response occurred several minutes after perfusion with PGE_2 _had begun and lasted approximately 25 minutes beyond the point when perfusion with PGE_2 _was stopped. Similar changes were recorded for the other warm sensitive neurons that were inhibited by PGE_2_, which showed response latencies of 3.90 ± 0.95 minutes and durations that ranged from 10 to 25 minutes.

Throughout the entire length of a recording (46.7 ± 1.8 minutes), the amplitude and duration of action potentials recorded from VMPO neurons slowly decreased by an average of 4.6 mV and 0.16 milliseconds, respectively (n = 42). It is presumed that this was due to minor changes in ionic gradients, resulting from the technique of whole-cell recording. Based on the PGE_2 _dependent changes in firing rate reported in this study and previous extracellular recordings [12], these small changes in action potential amplitude and duration did not affect the ability of VMPO neurons to respond to PGE_2_. There were also no significant changes in the amplitudes or durations of action potentials recorded from temperature insensitive and warm sensitive neurons in response to PGE_2_.

**Figure 3:**
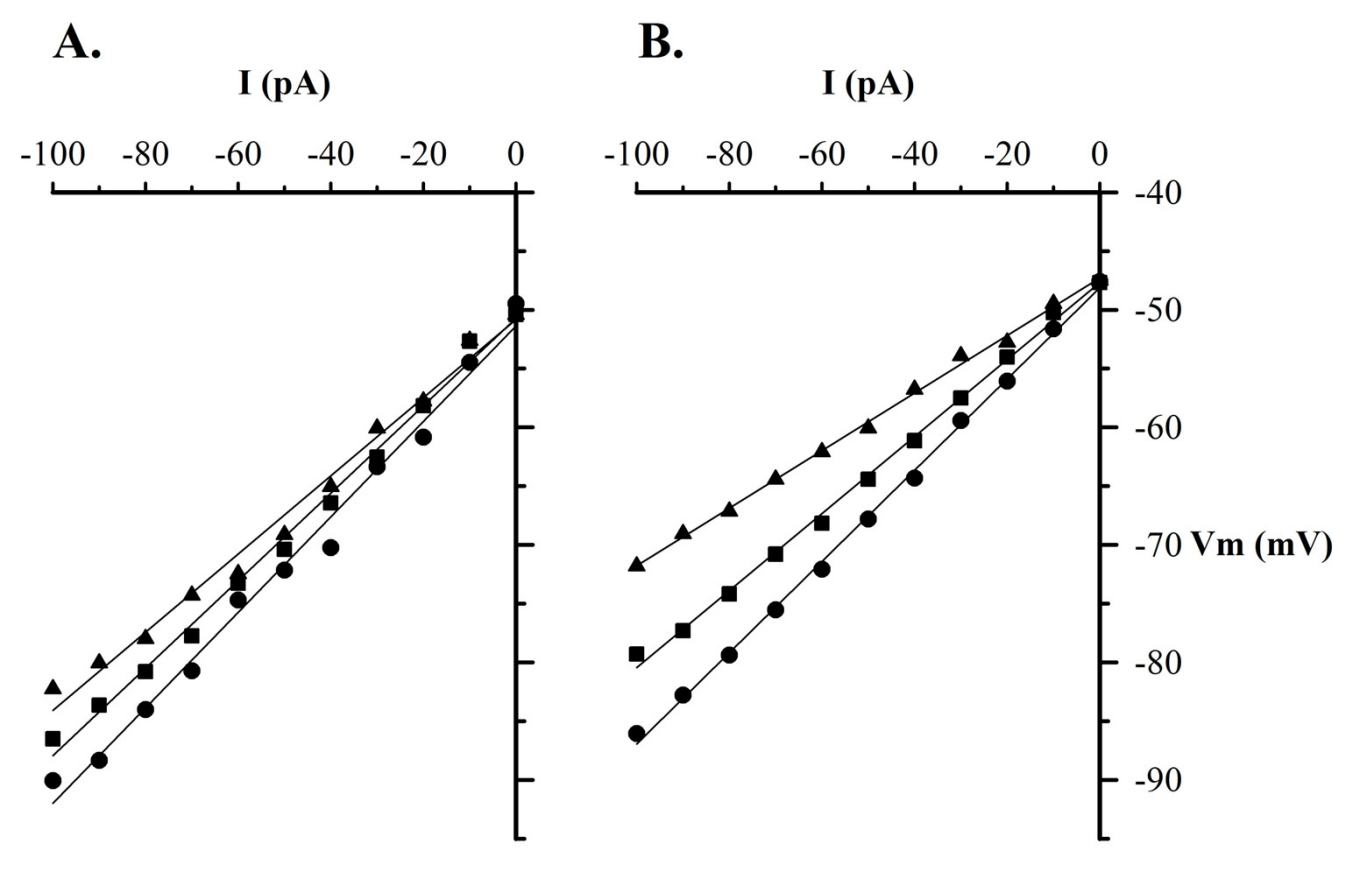
**The effects of PGE_2 _on the input resistance of a VMPO temperature insensitive neuron (A) and a VMPO warm sensitive neuron (B). **For each, changes in membrane potential are plotted in response to a series of hyperpolarizing current pulses (-10 to -100 pA, 200 ms), during baseline conditions (●), perfusion with PGE_2 _(▲), and after PGE_2 _has been washed out of the chamber (■). For the temperature insensitive neuron in **A **(m = 0.05), input resistance decreased from a baseline of 406.33 MΩ, to 332.40 MΩ during perfusion with PGE_2_. After PGE_2 _had been washed out of the chamber, input resistance increased to 372.32 MΩ. For the warm sensitive neuron in B (m = 1.30), input resistance decreased from a baseline of 388.00 MΩ, to 244.86 MΩ during perfusion with PGE_2_. After PGE_2 _had been washed out of the chamber, input resistance increased to 327.63 MΩ.

The input resistance of VMPO neurons recorded in this study significantly decreased in response to PGE_2 _from 367.1 ± 24.5 MΩ. to 339.7 ± 23.7 MΩ (n = 42; paired T test, P = 0.0001). This PGE_2 _dependent decrease in resistance was independent of thermosensitivity. Figure 3 shows current-voltage plots from a temperature insensitive neuron (Fig. 3A) and a warm sensitive neuron (Fig. 3B). For both of these neurons, input resistance decreased similarly in response to PGE_2_, with a reversal potential at or near the resting membrane potential.

**Figure 4:**
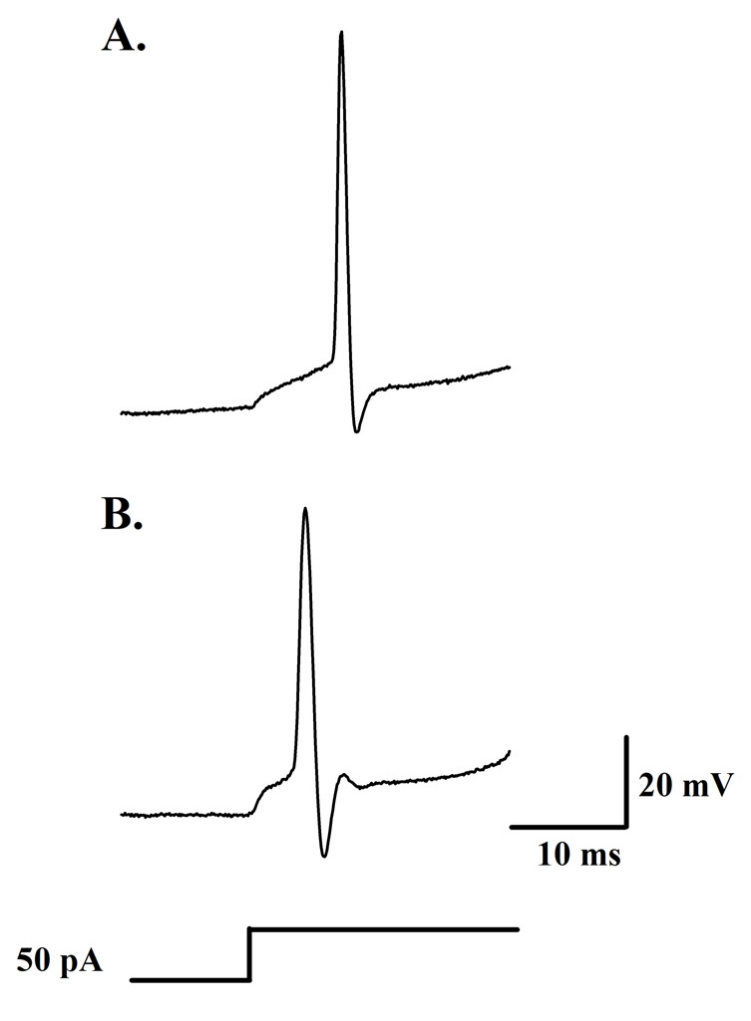
**The effect of depolarizing current and PGE_2 _on the activity of a VMPO temperature insensitive neuron. A **shows the initial action potential that was produced during baseline conditions, in response to a depolarizing current injection (50 pA, 500 msec). The onset of the current is indicated on the line at the bottom of the figure and only the initial 34 mSec are shown. The first spike latency was 8.0 mSec. In **B**, the initial action potential that was produced in response to a depolarizing current injection is shown during perfusion with PGE_2 _(1 μM). The first spike latency was 3.0 mSec.

**Figure 5:**
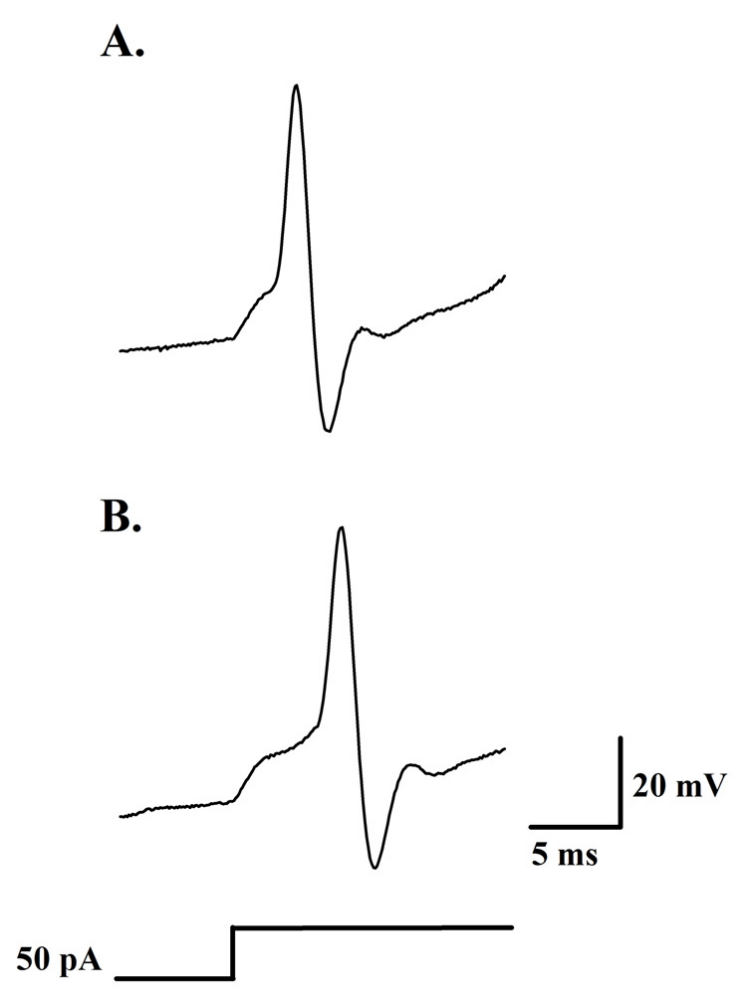
**The effect of depolarizing current and PGE_2 _on the activity of a VMPO warm sensitive neuron. A **shows the initial action potential that was produced during baseline conditions, in response to a depolarizing current injection (50 pA, 500 msec). The onset of the current is indicated on the line at the bottom of the figure and only the initial 22.0 mSec are shown. The first spike latency was 4.0 mSec. In **B**, the initial action potential that was produced in response to a depolarizing current injection is shown during perfusion with PGE_2 _(200 nM). The first spike latency was 6.6 mSec.

During the application of a depolarizing current, VMPO neurons did not show PGE_2 _dependent changes in the frequency of action potentials (frequency response). However, the majority of VMPO neurons did show PGE_2 _dependent changes in another characteristic of neuronal excitability, the first spike latency. Temperature insensitive neurons with thermosensitivities ≤ 0.4 impulses·s^-1.^°C^-1^showed a significant PGE_2 _dependent decrease in the first spike latency, from 7.4 ± 1.2 ms during baseline conditions to 5.2 ± 0.6 ms in response to PGE_2 _(n = 18; paired T test, P = 0.008; Figure 4). The first spike latency for these temperature insensitive neurons returned to baseline levels during the washout period (7.6 ± 1.4 ms). In contrast, warm sensitive neurons showed a significant increase in this latency response during perfusion with PGE_2_(n = 7), similar to the response of the warm sensitive neuron in Figure 5. (baseline = 4.9 ± 0.5 ms; PGE_2 _= 7.9 ± 1.1 ms (paired T test, P = 0.046); washout = 4.2 ± 0.5 ms). Temperature insensitive neurons with thermal coefficients of 0.41 to 0.79 impulses·s^-1.^°C^-1 ^showed little change in this measurement of neuronal excitability (n = 8), in response to PGE_2 _(baseline = 7.0 ± 1.3 ms; PGE_2 _= 6.8 ± 1.1 ms (paired T test, P = 0.69); washout = 6.8 ± 1.1 ms).

**Figure 6:**
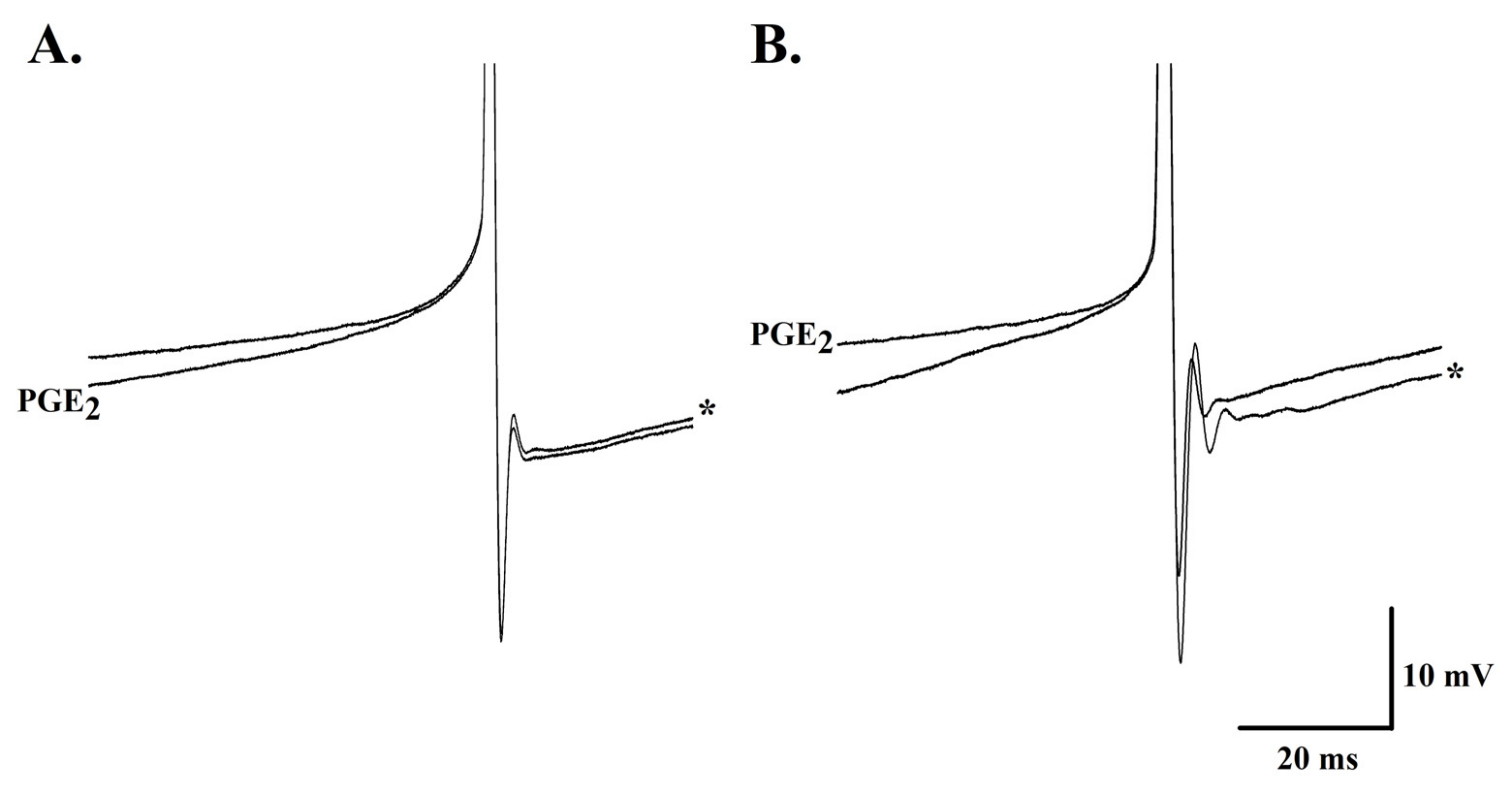
**Averaged pre- and post-spike activity of a temperature insensitive VMPO neuron (A) and warm sensitive VMPO neuron (B)**. For each, averages of 10 action potentials (truncated) during baseline conditions and during perfusion with PGE_2 _(1 μM), are superimposed on the spike threshold (post-pike activity during PGE_2 _is indicated by an asterisk). These averages do not include pre-spike activity that contained putative postsynaptic potentials. For the temperature insensitive neuron in **A**, the rate of rise of the depolarizing prepotential increased from 0.17 mV·mSec^-1 ^to 0.27 mV·mSec^-1^. For the warm sensitive neurons in **B**, the rate of rise of the depolarizing prepotential decreased from 0.38 mV·mSec^-1 ^to 0.18 mV·mSec^-1^.

In Figure 6, the averaged pre- and post-spike activity of a temperature insensitive neuron (A) and a warm sensitive neuron (B) are shown during baseline conditions and in response to PGE_2_. In a similar manner to the neuron shown in Figure 6A, the majority of temperature insensitive neurons with thermal coefficients ≤ 0.4 impulses·s^-1.^°C^-1 ^showed a significant increase in the rate of rise of the depolarizing prepotential in response to PGE_2 _(n = 12; baseline = 0.46 ± 0.04 mV·ms^-1^, PGE_2 _= 0.55 ± 0.05 mV·ms^-1^; paired T test, P = 0.01). In contrast, the majority of warm sensitive neurons showed a significant decrease in the rate of rise of the depolarizing prepotential in response to PGE_2_, similar to the response of the neuron in Figure 6B (n = 8; baseline= 0.61 ± 0.08 mV·ms^-1^, PGE_2 _= 0.50 ± 0.08 mV·ms^-1^; paired T test, P = 0.012). Although small changes in the voltage deflections that occurred at the end of an action potential showed some degree of change in response to PGE_2_, these changes were inconsistent in both temperature insensitive or warm sensitive neurons. Temperature insensitive neurons with thermosensitivities of 0.41 – 0.79 impulses·s^-1.^°C^-1 ^did not show a significant change in the rate of rise of the depolarizing prepotential in response to PGE_2_.

### Synaptic input

The frequency of post synaptic potentials (PSPs) recorded from VMPO neurons in the localized environment of coronal tissue slices was predominately inhibitory and insensitive to changes in temperature (Table 1). In 92% of the recordings, temperature had little or no effect on the frequencies of either inhibitory post synaptic potentials (IPSPs; m = 0.25 ± 0.06 PSPs·s^-1.^°C^-1^) or excitatory post synaptic potentials (EPSPs; m = 0.23 ± 0.06 PSPs·s^-1.^°C^-1^). In response to PGE_2_, the frequency of synaptic input recorded from VMPO neurons did not change (Table 1).

## Discussion

### PGE_2 _dependent changes in the firing rates of VMPO neurons

As current models for temperature regulation suggest, the integrated responses of hypothalamic warm sensitive neurons and temperature insensitive neurons play an important role achieving and maintaining a discrete set-point for temperature control [2]. While their importance in thermoregulatory pathways has yet to be determined, *in vitro *recordings from tissue slices have also identified neurons in the PO/AH that lack spontaneously generated activity (silent neurons), are predominantly driven to produce action potentials by synaptic input (EPSP-driven neurons), or produce action potentials in a bursting pattern [2,15]. However, only prepotential driven warm sensitive and temperature insensitive neurons were identified in the VMPO.

In the generation of a fever, current thermoregulatory models of neural networking suggest that either an increase in the activity of temperature insensitive neurons or the inhibition of warm sensitive neurons would shift the set-point to a more hyperthermic temperature [2]. Through competing synaptic inputs, either of these responses would alter the temperature at which thermoeffector neurons begin to activate thermoregulatory responses. However, previous studies have not been able to show a correlation between the thermosensitivity of hypothalamic neurons and firing rate responses to PGE_2 _or other endogenous pyrogens [16-19]. This may be the result of the various criteria that were used to define thermosensitivity in many of these studies, as well as recordings from more general areas of the PO/AH. In a recent study of the extracellular single-unit activity of neurons in the VMPO, a clear thermal distinction in firing rate responses to PGE_2 _was reported, with temperature insensitive neurons excited by PGE_2 _and warm sensitive neurons inhibited [12]. In this previous study, a functionally significant criterion was used to define warm sensitivity (m ≥ 0.8 impulses·s^-1.^°C^-1^) [3,15]. As this criterion provides a method for identifying integrative warm sensitive neurons in the tissue slice preparation, it allows the experimental findings to have a functional significance in modeling thermoregulation *in vivo*. In the present study, which uses whole-cell recording techniques to record the intracellular properties of VMPO neurons and the same functional criterion for defining warm sensitivity, similar firing rate responses were recorded. As suggested above, either an increase in the firing rates of temperature insensitive neurons or a decrease in the firing rates of warm sensitive neurons could lead to a hyperthermic shift in the thermostatic set-point and a fever.

It is also important to note that while the majority of PGE_2 _responsive neurons showed a significant recovery in firing rate during the washout period, some did not. This lack of complete recovery may have resulted from responses to PGE_2 _that were considerably long in duration, lasting up to 40 minutes, and minor changes in ionic gradients that can occur over time during whole-cell recordings. As mentioned in the methods, all recordings were closely monitored for any changes that would make them unacceptable.

### Cellular properties of VMPO neurons and responses to PGE_2_

In addition to the direct effects of PGE_2 _on the electrical properties of VMPO neurons, the frequency of localized synaptic input was characterized. In the present study, we were specifically interested in monitoring firing rate responses to PGE_2 _and characterizing the resulting voltage potential changes (i.e., action potentials and membrane potentials). In order to ensure that we did not interrupt the firing rate responses, we did not perform any voltage clamp measurements and did all recordings in current clamp, with current = 0 pA. This prevented any detailed characterization of synaptic potentials, other than frequency. As has been shown in other regions of the PO/AH [15], the majority of this synaptic input to VMPO neurons was inhibitory and predominantly insensitive to temperature. In response to PGE_2_, the frequency of PSPs recorded from most VMPO neurons either did not change or showed an increase. However, PGE_2 _dependent increases in the frequency of PSPs were not recorded from neurons that showed PGE_2 _dependent changes in firing rate. This may have resulted from a smaller degree of synaptic influence on firing rate, due to a change in the input resistance [20]. Additionally, the lack of local synaptic input from warm sensitive neurons would suggest that the axonal projections of these neurons may not terminate within the VMPO, but form efferent projections to other hypothalamic nuclei such as the paraventricular nucleus.

As shown in Figure 3, the input resistance recorded from most of the VMPO neurons decreased in response to PGE_2_, regardless of temperature sensitivity or PGE_2 _dependent changes in firing rate. Since this response had little or no influence on firing rate, we would suggest that decreases in resistance resulted from changes in the activity of multiple currents, which continued to maintain equilibrium at or near the resting membrane potential. While we have also suggested that PGE_2 _may cause cAMP concentrations to either increase or decrease within these neurons [12,21], the net effect on hyperpolarizing K^+ ^currents and depolarizing Na^+ ^or Ca^++ ^currents may still lead to an increase in conductance that does not result in a change in resting membrane potential. Therefore, although these changes in the input resistance may be an important response to PGE_2_, they are not directly responsible for PGE_2 _dependent changes in firing rate.

In response to a depolarizing current pulse, PGE_2 _dependent changes in the excitability of VMPO neurons were recorded. Using two primary measurements of excitability, the frequency response and first spike latency (see: Results & Methods), our recordings indicate that PGE_2 _selectively increased the excitability of temperature insensitive neurons, while decreasing the excitability of warm sensitive neurons. Although there was no significant PGE_2 _dependent changes in the frequency responses of VMPO neurons, temperature insensitive neurons showed a significant decrease in first spike latency in response to PGE_2 _(Fig. 4), while warm sensitive neurons showed a significant increase (Fig. 5). This would suggest that PGE_2 _is having a direct effect of the activity of transient voltage dependent currents, which are responsible for regulating the excitability and rhythmic firing rates of these neurons. While these current pulses were not matched so that they would result in the same level of depolarization, making it difficult to discern which voltage-gated conductances may be responding to PGE_2_, this data does provide support for our finding of PGE_2 _dependent changes in the prepotential [22].

Several transient voltage dependent currents have been identified in hypothalamic neurons which regulate excitability and rhythmic firing rate activity. These include a slow component sodium current and a calcium dependent potassium current (I_K, Ca_) in suprachiasmatic neurons [23], a low voltage activated calcium current and a I_K, Ca _current in posterior hypothalamic neurons [24], a non-inactivating potassium current in neurhypophyseal nerve terminals [25], and a I_A _current in PO/AH neurons [22]. Although any or all of these currents may be present in VMPO neurons, only the I_A _has been implicated in temperature dependent changes in firing rate, through a mechanism in which the thermally dependent inactivation rate of this current influences the rate of rise of the prepotential that precedes each action potential [22]. In a similar manner, our data suggests that PGE_2 _has a direct effect on the firing rates of VMPO neurons through changes in the prepotential, increasing the rate of rise in temperature insensitive neurons, while decreasing the rate of rise of the prepotential in warm sensitive neurons (Fig. 6). Therefore, PGE_2 _dependent changes in the firing rates of VMPO neurons may also depend on an ability to influence the inactivation rate of the I_A _type current.

Within the VMPO, overlapping expression of EP_3 _and EP_4 _receptors may provide PGE_2 _with the ability to selectively affect the activity of neurons in this region [26]. Activation of either receptor subtype is known to influence cellular activity through the regulation of intracellular cAMP concentrations, with EP_4 _activation leading to an increase in cAMP and EP_3 _activation leading to a decrease [26-28]. Support for this mechanism is provided by evidence showing that cAMP plays a role in thermoregulation and more specifically, in the generation of a fever [29]. With respect to excitability and rhythmic firing rate activity, several studies have demonstrated that cAMP modulates PGE_2 _dependent changes the activity of certain potassium currents, including the I_A _[25,30,31]. Therefore, selective activation of either EP_3 _or EP_4 _receptors may be responsible for the changes in firing rate and electrical activity we have recorded from VMPO neurons.

## Conclusion

In response to intravenous LPS, the local production of PGE_2 _within the PO/AH region results in a thermally dependent change in the firing rates of VMPO neurons. Through a direct effect on the rate of rise of the depolarizing prepotential, which is also a determinant of thermosensitivity, PGE_2 _increases the firing rates of temperature insensitive neurons, while decreasing the firing rates of warm sensitive neurons in the VMPO. While the results of this study provide a clear focus for future studies into the conductance mechanisms of these responses, it also supports a functional and important role for the VMPO region and both groups of thermally classified neurons in the production of this type of fever.

## Methods

Anterior hypothalamic tissue slices containing the VMPO were prepared from male Sprague-Dawley rats (100 – 150 grams), which were housed under standard conditions and given food and water *ad lib*. Prior to each recording session, an animal was anesthetized (isoflurane) and sacrificed by quick decapitation, according to procedures approved by the National Science Foundation and the Animal Care and Use Committee of the College of William and Mary. Following removal of the brain, a tissue block of the hypothalamus was cut using a vibratome into 400 μm thick coronal slices. Two or three slices containing the VMPO were then placed in a recording chamber and allowed to equilibrate for 1–2 hours. Tissue slices were continually perfused with pyrogen free artificial cerebral spinal fluid (aCSF), which consisted of (in mM), 124 NaCl, 26 NaHCO_3_, 10 glucose, 5 KCl, 2.4 CaCl_2_, 1.3 MgSO_4_, and 1.24 KH_2_PO_4_. This nutrient medium was oxygenated (95% O_2 _– 5% CO_2_), warmed to a stable temperature of ~36°C by a thermoelectric assembly, and allowed to gravity flow into the chamber at 1 – 1.5 ml· min^-1 ^(chamber volume = 1.2 ml) [32]. A small thermocouple, positioned in the recording chamber just below the tissue slices, was used to continuously monitor tissue temperature.

Tight-seal whole-cell recordings were made using glass microelectrodes with tip inner diameters of ~2 μm (3–5 MΩ), filled with a solution that consisted of (in mM) 130 K-gluconate, 10 EGTA, 2 ATP, 1 MgCl_2_, 1 CaCl, having a pH of 7.2 and an osmolarity of approximately 295 mOsmols/liter. As described previously [33], a liquid junction potential of 12.0 mV was subtracted from all recorded potentials. All recordings were made using an integrated patch-clamp amplifier (Axopatch 200B, Axon Instruments) and along with temperature, were stored on digital tape for later analysis. Firing rate was continuously recorded as a voltage measurement from a rate/interval monitor and was determined by action potential triggered input from a window discriminator (FHC Inc.). Specific stimulation protocols to measure input resistance and excitability were generated by a computer that was interfaced with the amplifier (pClamp software, Axon Instruments). Acceptable recordings consisted of action potential amplitudes through 0 mV and stable recordings of at least 20 minutes.

Using a stereomicroscope, the recording electrode was positioned in the VMPO, which encompasses 700 μm of the hypothalamus just rostral to the suprachiasmatic nucleus. It extends laterally 900 μm from the third ventricle and dorsally 500 μm from the ventral brain surface [34]. Once a tight seal (> 2 GΩ) was achieved between the electrode and the surface of a neuron, the cell membrane was ruptured by suction, establishing an intracellular recording. When the activity of a neuron was stable for several minutes, temperature in the recording chamber was varied 2–3°C above and below 36°C, by changing the input voltage to the thermoelectric assembly. Neuronal thermosensitivity (impulses· s^-1^·°C^-1^) was characterized by plotting firing rate as a function of temperature to determine the regression coefficient (m) of this plot. As in previous studies [12,15,35], warm sensitivity was defined as a regression coefficient of at least 0.8 impulses· s^-1^·°C^-1^. All other neurons in this study were defined as temperature insensitive.

After thermosensitivity had been characterized, each neuron was tested for its response to PGE_2_. At a stable temperature (~36°C), the perfusion medium was switched to one containing PGE_2 _(200 nM or 1 μM, Sigma Chemical Co.). The duration of exposure to PGE_2 _ranged from 5 – 15 minutes, with durations of less than 10 minutes occurring only when there was a clear indication of a response. Exposure to PGE_2 _was followed by perfusion with aCSF for a washout period of at least 10 minutes.

To determine if PGE_2 _had a significant effect on firing rate, one-minute segments of stable activity were digitized for comparison (60 Hz; pClamp Software, Axon Instruments). These segments were collected during baseline conditions (just prior to perfusion with PGE_2_) at the end of perfusion with PGE_2_, and at the end of a 10-minute washout period (or when firing rate returned to baseline levels). For each segment, a mean and standard error were calculated (Sigmaplot software, SPSS Inc.). A significant response to PGE_2 _was determined by comparison to the baseline level using a standard T-test (P ≤ 0.05).

Changes in resting membrane potential and the action potential waveform in response to PGE_2 _were also characterized. As in a previous study [32], the resting membrane potential was continuously recorded. After rapid changes in membrane potential (including action potentials) were filtered out (half amplitude response setting at 0.5 Hz), one minute segments of voltage activity were digitized (60 Hz) during baseline conditions, perfusion with PGE_2_, and the washout period. To characterize changes in the action potential, an averaged action potential from ten inherently activated action potentials was produced for each of the experimental conditions (sampling rate = 66.7 kHz). Measurements were made of action potential amplitude (resting membrane potential to peak) and duration (at one half peak). Measurements were also made of the rate of rise of the depolarizing prepotential that precedes each action potential [22]. For resting membrane potential and action potential measurements, significant responses to PGE_2 _were determined by comparison to baseline using a standard T-test (P ≤ 0.05).

Throughout each recording, input resistance and excitability were measured every 5 – 10 minutes to determine changes in response to PGE_2 _and to insure that recordings remained stable. Large changes that did not return towards baseline levels were a characteristic of a deteriorating recording and marked the end of legitimate data. Input resistance was determined by the slope of a current-voltage plot obtained from a computer generated protocol in which a series of ten current pulses (-10 to -100 pA) were administered [32]. In addition, two primary characteristics of neuronal excitability were measured in response to a depolarizing current [36]. The frequency response was determined by the number of action potentials produced during the depolarizing current. The first spike latency was also measured and was defined as the duration from the start of the depolarizing current to the peak of the first action potential. All significant responses to PGE_2 _were again determined by comparison to baseline using a standard T-test (P ≤ 0.05).

The frequencies of synaptic potentials (EPSPs and IPSPs) recorded from each VMPO neuron were determined during baseline conditions, perfusion with PGE_2_, and the washout period. As in previous whole-cell recordings, individual potentials were identified as rapid changes in membrane potential of at least 1 mV greater than background noise [15,20]. For each experimental condition, blind counts of PSPs were made over a 20 second duration to produce frequency averages (PSPs·s^-1^). As with the other measurements, all significant responses to PGE_2 _were determined by comparison to baseline using a standard T-test (P ≤ 0.05). In addition, the thermosensitivities of EPSPs and IPSPs were characterized by calculating frequency averages (using the same methods as detailed above) at different temperatures (≥ 3°C range) and plotting the results as a function of temperature [15].

Once a recording had been completed, a stereomicroscope was used to visually confirm the location of the recording electrode. The ventral edge of the third ventricle was used as a reference to determine the lateral-medial and dorsal-ventral coordinates. The coronal position was also specified by the depth of the electrode from the surface of the tissue slice. In addition, the side of the brain from which the recording was made was identified by preparing the tissue slices with more lateral tissue on the left. Tissue slices were then removed from the recording chamber, fixed in a 10% formalin solution, and sectioned again to a thickness of 50 μm. Sections were then stained with giemsa to identify specific hypothalamic areas so that the location of each recording within VMPO could be reconfirmed [12] [38].

## Authors' contributions

HJR, carried out the majority of cellular recordings and data analysis. JDG conceived of the study and participated in its design, coordination and completion. Both authors contributed equally to the drafting of this manuscript.

## Figures and Tables

**Table 1:** Effect of PGE_2_on the Frequency of IPSPs and EPSPs recorded from VMPO Neurons.

		IPSPs·s^-1^			EPSPs·s^-1^		
			
**Thermosensitivity (impulses·s^-1.^°C^-1^)**	**N**	**Baseline**	**PGE_2_**	**Washout**	**Baseline**	**PGE_2_**	**Washout**
≤0.4	19	5.1 ± 1.2	5.6 ± 1.0	5.3 ± 1.0	3.0 ± 0.0	3.1 ± 0.7	3.2 ± 0.1
0.41 – 0.79	13	6.4 ± 1.1	7.3 ± 1.3	6.2 ± 1.1	3.3 ± 0.7	4.2 ± 3.3	3.7 ± 0.8
≥ 0.8	10	4.2 ± 1.1	3.8 ± 0.9	3.8 ± 0.9	2.4 ± 0.7	3.2 ± 0.4	1.4 ± 0.3
